# How machine learning can assist the interpretation of *ab initio* molecular dynamics simulations and conceptual understanding of chemistry†
†Electronic supplementary information (ESI) available: Brief introduction to PCA. Further details on the implementation of the BNN models and on the hyperparameters optimisation including the scanned hyperparameter and obtained performances of different BNN architectures. Analysis of the BNN performance with the training set size. Predictions of dissociation half-times for vibrational states excited along two normal modes. Dynamics simulations of the dissociation of 1,2-dioxetane for different vibrational ground and excited states. Transition state structure and normal modes of the unmethylated 1,2-dioxetane. The two trained BNN can be found here: https://github.com/FlorianHase/LearningMoleculeDissociations. See DOI: 10.1039/c8sc04516j


**DOI:** 10.1039/c8sc04516j

**Published:** 2018-12-21

**Authors:** Florian Häse, Ignacio Fdez. Galván, Alán Aspuru-Guzik, Roland Lindh, Morgane Vacher

**Affiliations:** a Department of Chemistry and Chemical Biology , Harvard University , Cambridge , Massachusetts 02138 , USA; b Department of Chemistry – Ångström , The Theoretical Chemistry Programme , Uppsala University , Box 538 , 751 21 Uppsala , Sweden . Email: morgane.vacher@kemi.uu.se; c Department of Chemistry and Department of Computer Science , University of Toronto , Toronto , Ontario M5S 3H6 , Canada; d Vector Institute for Artificial Intelligence , Toronto , Ontario M5S 1M1 , Canada; e Canadian Institute for Advanced Research (CIFAR), Senior Fellow , Toronto , Ontario M5S 1M1 , Canada

## Abstract

Molecular dynamics simulations are often key to the understanding of the mechanism, rate and yield of chemical reactions. One current challenge is the in-depth analysis of the large amount of data produced by the simulations, in order to produce valuable insight and general trends. In the present study, we propose to employ recent machine learning analysis tools to extract relevant information from simulation data without *a priori* knowledge on chemical reactions. This is demonstrated by training machine learning models to predict directly a specific outcome quantity of *ab initio* molecular dynamics simulations – the timescale of the decomposition of 1,2-dioxetane. The machine learning models accurately reproduce the dissociation time of the compound. Keeping the aim of gaining physical insight, it is demonstrated that, in order to make accurate predictions, the models evidence empirical rules that are, today, part of the common chemical knowledge. This opens the way for conceptual breakthroughs in chemistry where machine analysis would provide a source of inspiration to humans.

## Abstract 2

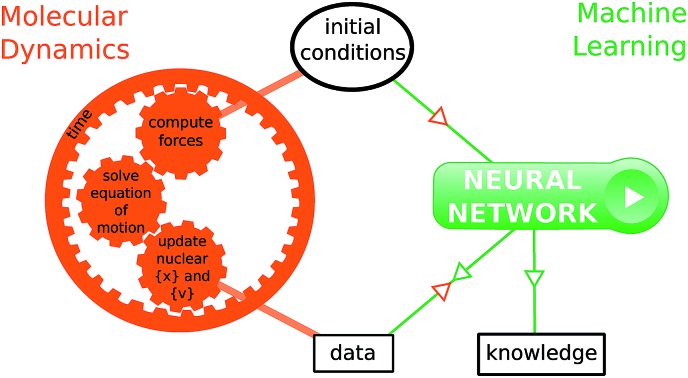
Machine learning models, trained to reproduce molecular dynamics results, help interpreting simulations and extracting new understanding of chemistry.

## 1 Introduction

Computer simulations are a key complement to experiments in the laboratory, especially when the latter are expensive or challenging because of extreme conditions required. Simulations also provide much greater details of a molecular process than can be observed experimentally. For instance, studying the time evolution of matter with molecular dynamics simulations is essential for understanding the mechanism, rate and yield of chemical reactions. Such simulations are also necessary to complement experiments and connect with time-resolved pump-probe measurements. At each time step of an *ab initio* molecular dynamics simulation, the energies and forces felt by the nuclei are calculated “on-the-fly” with an electronic structure method. With the growing complexity of the investigated chemical problems and the increasing need for improved accuracy, molecular dynamics simulations become very time-consuming. Typical time and length scales that are accessible with *ab initio* molecular dynamics are, with current computer systems, up to hundreds of femtoseconds (fs) to tens of picoseconds, and tens to few hundreds of atoms. As simulations become more complex, their usefulness for guidance and understanding may become obscured. Simple lessons are often lost among gigabytes or terabytes of data. The present work proposes to use machine learning methods to aid the interpretation of molecular dynamics simulations. The bigger goal here is in the future to allow machines to provide a source of inspiration to humans for the elaboration of new concepts in chemistry. This has been identified by some of us as one of the six grand challenges for the simulation of matter in the 21st century^1^ and the present work demonstrates an approach for achieving this.

**Fig. 1:**
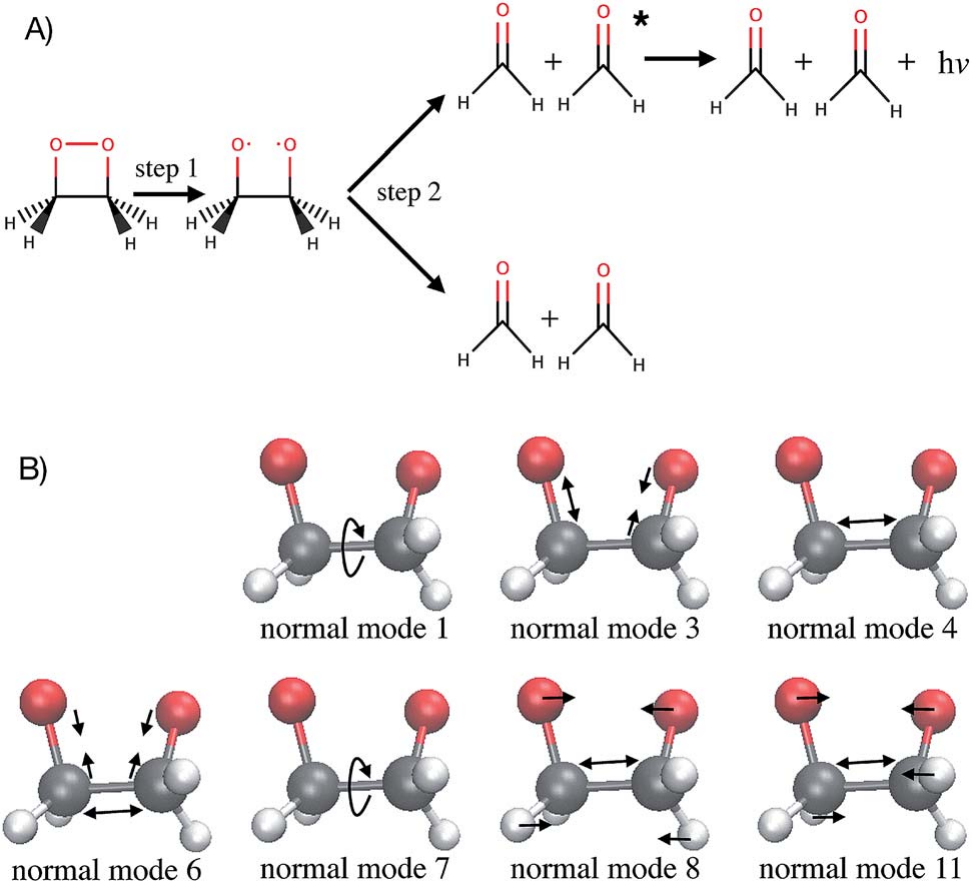
1,2-Dioxetane molecule. (A) Dark and chemiluminescent dissociation reactions. (B) Schematic representation of the relevant normal modes. Normal mode 1 corresponds mainly to the OCCO dihedral angle while normal mode 7 corresponds mainly to the HCCH dihedral angles.

As a test application, the timescale of the chemiluminescent decomposition of 1,2-dioxetane is chosen (Fig. 1A). Chemiluminescence is the emission of light as a result of a chemical reaction. This process is called bioluminescence when occurring in living organisms as in the well-known example of the firefly. For a recent review on the topic, see ref. 2 and 3. Chemiluminescence and bioluminescence are increasingly used in biological and chemical analysis methods,^4^ in various fields such as DNA sequencing,^5^ immunoassays as an alternative to radioactive isotopes^6^ and as a sensitive probe for mechanical stimulations.^7^,^8^ The applications are of course limited by the light emission efficiency, *i.e.* the chemiluminescence yield. 1,2-dioxetane is known to be the smallest compound with chemiluminescent properties. Upon thermal activation, it decomposes into two formaldehyde molecules in a two-step process: first the O–O bond breaks (Fig. 1A, step 1) and then the C–C bond (Fig. 1A, step 2).^9^,^10^ Non-adiabatic transitions from the electronic ground state to electronic excited states during the decomposition reaction lead to chemiexcitation. The resulting electronic excited states can relax back to the electronic ground state by emitting light. Previous *ab initio* molecular dynamics simulations have been performed for several methyl-substituted dioxetane molecules.^11^,^12^ Those simulations have shown that the chemiexcitation yield is determined by the dissociation timescale: the longer the molecule stays in the so-called “entropic trap” region before dissociating, the higher the chemiexcitation yield. Being able to calculate and understand the dissociation time of dioxetanes is thus essential for rationalising chemiluminescence yields measured experimentally and for designing new and efficient chemiluminescent compounds. It is, however, *a priori* unclear which molecular modifications would eventually result in significant changes of the dissociation time. Testing possible hypotheses with *ab initio* methods is an arduous process due to the computational demand required by the methods and can easily exceed available computing resources.

In the present work we illustrate how machine learning models can be used to solve this task. More specifically, we employ Bayesian neural networks (BNN) which are trained to predict the dissociation times of 1,2-dioxetane from initial nuclear positions (or from initial nuclear positions and velocities). We demonstrate that the trained BNN can be used in two different ways: (i) to autonomously find physical correlations, and (ii) to test already formulated hypotheses. This is performed on both the unmethylated and tetramethylated dioxetanes, the latter presenting a longer dissociation timescale and thus a higher chemiexcitation yield than the former. High prediction accuracy for the dissociation time is achieved despite the limited size of the dataset, which consists of 250 trajectories simulating up to 250 fs with a time step of 0.24 fs. This presents an advancement over prior studies with datasets with orders of magnitude more data points.^13^,^14^ More importantly, we provide an example for how to interpret the machine learning model itself and the changes it has undergone during the training procedure in order to learn how to reproduce the targeted outcome. In addition, the trained BNN is used to predict dissociation times of vibrationally excited states of the unsubstituted 1,2-dioxetane. Detailed understanding of the effect of specific nuclear distortions, that could be either enhanced or diminished *via* chemical modification, opens up possibilities for more efficient molecular design. Still aiming at gaining physical insight, it is demonstrated that while being trained to reproduce the dissociation times of 1,2-dioxetanes with high accuracy, the BNN has evidenced some chemical rules that connect the nuclear positions (and velocities) and the dissociation times.

It is noted that to reduce the computational cost of molecular dynamics simulations, a lot of efforts have recently been devoted to construct efficient models that “learn” or fit the potential energy surfaces many orders of magnitude faster than electronic structure calculations.^13^,^15^–^22^ For example, machine learning techniques have been used to reproduce energies at the level of hybrid density-functional theory, from lower-level calculations and so-called molecular descriptors^23^,^24^ or directly from just the Cartesian coordinates and the nuclear charges.^25^–^27^ The present work goes to a higher level of abstraction and proposes to use a machine learning model to directly predict a specific outcome of the molecular dynamics simulation, bypassing the construction of potential energy surfaces and avoiding the computation of the time propagation. The focus here is however neither on reducing the computational cost of electronic structure in *ab initio* molecular dynamics simulations, nor on describing with quantitative accuracy chemiluminescent or bioluminescent reactions. It is rather on training a machine learning model on already simulated trajectories (of a given chemical reaction and at a given level of theory), and interpreting and using the trained machine learning model in order to gain physical insight into the studied chemical reaction. We describe two possible approaches to gain insights: studying the trained models themselves to find correlations, and using the models to test already formulated hypotheses. We note further that these approaches do not depend on the way the potential energy surfaces are generated along the dynamics trajectories. Our strategy could be used as well to interpret dynamics simulations based on machine-learned potential energy surfaces. In fact, the use of machine learning to reduce the cost of *ab initio* molecular dynamics simulations is expected to increase the amount of data generated and to be interpreted, which will further increase the need for the tools proposed in the present work.

## 2 Methods

### 2.1 *Ab initio* molecular dynamics simulations

The approach used for the *ab initio* molecular dynamics simulations is the same as in previously published works.^11^,^12^ The complete active space self-consistent field (CASSCF) method^28^,^29^ state-averaging over the four lowest-energy singlet states equally is used to describe the electronic structure of the system. The active space chosen consists of 12 electrons and 10 orbitals: the four σ and four σ* orbitals of the four-membered ring, plus the two oxygen lone-pair orbitals perpendicular to the ring. The ANO-RCC basis set with polarized triple-zeta contraction is used.^30^,^31^ Born–Oppenheimer dynamics is simulated with a time step of 10 au (≈0.24 fs) and all nuclear coordinates are taken into account. It is noted that, in the present simulations, only the electronic ground state is included and non-adiabatic transitions to electronic excited states are not allowed. The implementation of this method in the OpenMolcas package is used.^32^,^33^ The trajectories are initialized and propagated from the transition state for the O–O bond breaking – since the latter controls the overall reaction rate. A small amount of kinetic energy (1 kcal mol^–1^) is given along the reaction coordinate (as suggested in previous theoretical studies of post-transition state dynamics^34^) toward the biradical region where the O–O bond is broken. The Newton-X package^35^ was used to sample 250 initial positions and velocities along all normal modes (other than the reaction coordinate) from a Wigner distribution in order to reproduce the vibrational ground state. The normal modes were calculated at the transition state structure using the electronic structure method mentioned above. Dissociation is considered to occur when the C–C bond length exceeds 2.4 Å (two times the van der Waals radius of a carbon atom). It is noted that choosing a slightly smaller or larger value for the bond length dissociation threshold has been shown not to change any relative comparisons, nor the findings regarding the entropic trap and the effect of the singlet excited states.^11^

### 2.2 Machine learning predictions

**Fig. 2:**
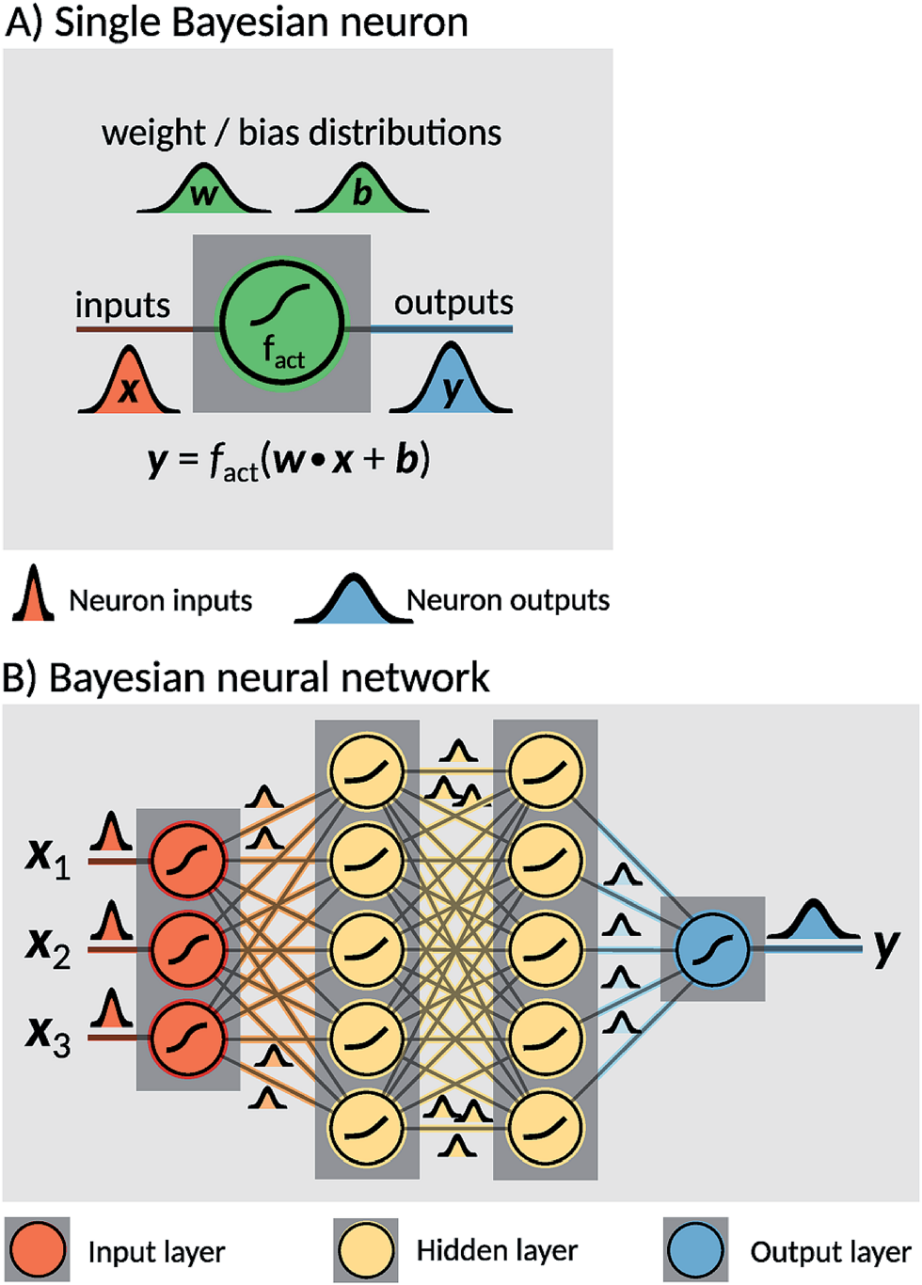
Illustration of a Bayesian Neural Network (BNN). (A): A Bayesian neuron defines a mathematical operation based on an activation function *f*_act_, a distribution of weights ***w*** and a distribution of biases ***b*** intrinsic to the neuron. Every input ***x*** is processed by sampling one instance of weights and biases from the distributions and applying the activation function. (B) A BNN consists of a set of interconnected Bayesian neurons. The neurons in the network are organised in layers, and can differ in their activation functions as well as their weight and bias distributions.

Two probabilistic models were used in order to estimate dissociation times of the dioxetane molecule: these are implemented as feedforward fully connected BNN. Neural networks are constructed as a set of nodes, called ‘neurons’, with connections between them (Fig. 2). Traditionally, each neuron is characterised by a weight ***w***, a bias ***b*** and an activation function *f*_act_. For a given input ***x***, a single neuron performs the operation

1 ***y*** = *f*_act_(***w***·***x*** + ***b***),

where ***y*** denotes the output of the neuron. The architecture of a neural network is defined by a set of parameters, collectively referred to as ‘hyperparameters’, which define for instance the number of neuron layers, the number of neurons per layer and the activation function of the neurons. In the case of a BNN, both weights ***w*** and biases ***b*** are modelled as random variables, sampled from a probability distribution, with conditional dependencies on either other model parameters or the input parameters, called ‘input features’ (Fig. 2A). As a consequence, a BNN produces an output distribution (Fig. 2B). The BNN can be trained so that the output distribution resembles a desired target distribution by adapting the distributions of weights and biases for each individual neuron. As such, BNN propagate information based on entire parameter distributions as opposed to traditional neural networks, which compute a target value based on single-value parameters. BNN therefore retain the flexibility of traditional neural networks, but provide a more robust framework for identifying relevant correlations between inputs and outputs, especially for small and medium sized datasets.^36^

The two models constructed in the present work use different sets of input features. The first model, hereafter noted ‘BNN1’, uses only the initial nuclear geometry of the molecule. The second model, hereafter noted ‘BNN2’, also uses the initial nuclear velocities in addition to the geometry. The nuclear geometry and velocities are given in normal mode coordinates to account for translational and rotational invariances. The normal modes used are the ones calculated at the transition state structure as indicated above. For the unmethylated dioxetane molecule consisting of 8 atoms, BNN1 has therefore 18 input features while BNN2 has 36 input features. For the tetramethylated dioxetane molecule consisting of 20 atoms, BNN1 and BNN2 have thus 54 and 108 input features, respectively.

Datasets for the BNN models were extracted from the *ab initio* molecular dynamics simulations by sampling every fifth frame (up to dissociation) along each of the 250 trajectories, resulting in a total of 11 650 frames and the corresponding dissociation times. We split the total set of data into a training set (80%, 9320 frames), a validation set (10%, 1165 frames) and a test set (10%, 1165 frames). Data for the test set were selected as random samples from the entire set and were not used for any part of the training procedure other than for reporting the out-of-sample prediction accuracy after all BNN models have been trained. An informative and diverse training set was assembled from the remaining 90% of the dataset *via* principal component analysis (PCA).^37^ (The reader is referred to ESI† for a brief introduction to PCA.) We selected the frames for the training set which are maximally separated in the reduced PCA space spanned by the most contributing principal components. This protocol has shown to improve prediction accuracies in the context of excitation transfer property predictions.^18^ The frames, which were not selected with this protocol, were used as the validation set.

The BNN used for the present study were implemented in the probabilistic programming library edward,^38^ and model parameters were updated *via* variational inference using the Adam optimisation algorithm.^39^ We parametrised the distributions of weights and biases as Laplace distributions. This choice is made in order to construct interpretable models. While the training set is used to optimise the model parameters ***w*** and ***b*** of a BNN model of a given architecture, the validation set serves as a benchmark to determine the best performing BNN architecture. For both BNN1 and BNN2, we conducted extensive hyperparameter searches to determine the best performing BNN models. The most accurate BNN model was selected as the model with the lowest prediction error on the validation set. We computed an upper bound on the overfitting error for both most accurate constructed models, BNN1 and BNN2, based on the Rademacher complexity^40^ for k-layered networks:^41^ it is 2.20 fs for BNN1 and 2.38 fs for BNN2. Details on the implementation of the BNN models and on the hyperparameter optimisation including the scanned hyperparameter and obtained performances of different BNN architectures, are reported in the ESI.† It is noted here that hyperparameters of BNN1 and BNN2 were found to be similar, despite the different number of input features. It is important to remember that the BNN are trained to learn the dissociation time of 1,2-dioxetane or tetramethyl-1,2-dioxetane from a particular set of initial conditions (nuclear positions, and velocities for BNN2) without *a priori* knowledge about the dynamics of the chemical reaction of interest.

## 3 Results

In this section, the machine learning predictions are used to study the mechanism of the dissociative reaction of 1,2-dioxetane. The BNN trained for the unmethylated dioxetane molecule is analysed in detail and unless explicitly stated otherwise, the results are presented for this compound. We begin our analysis with validating that the trained BNN are indeed capable of predicting dissociation times within reasonable accuracy. Then, we proceed with analysing the architecture of the trained BNN to identify nuclear coordinates relevant to the dissociation timescale. Finally, we use the BNN to test hypotheses about physical correlations.

### 3.1 Validation of the dissociation time predictions

**Fig. 3:**
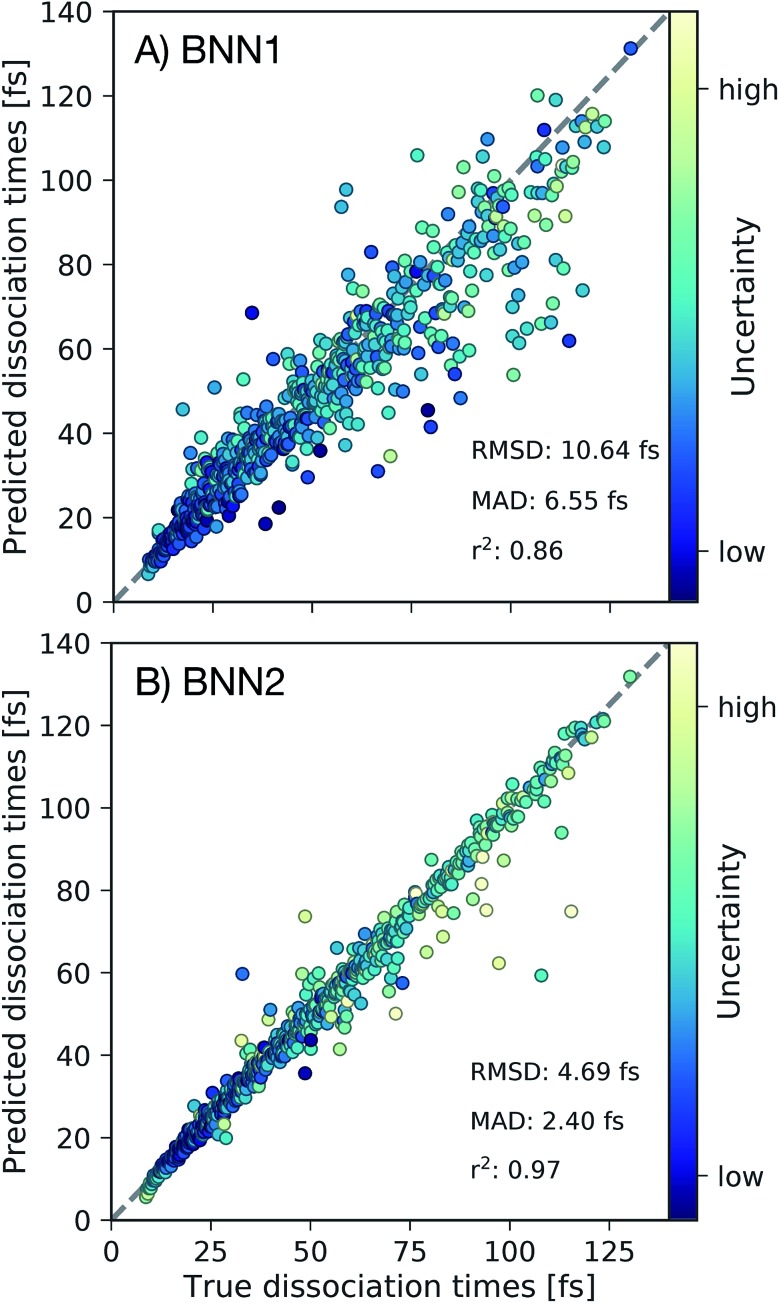
Out-of-sample predictions for best performing (A) BNN1 and (B) BNN2 models. For all frames in the test set (1165 frames) we depict the dissociation times predicted by the BNN models in comparison to the true dissociation times. Root mean square deviations (RMSD), mean absolute deviations (MAD) and coefficients of determination (*r*^2^) are reported. Depicted points are coloured based on the predicted uncertainty of the BNN model. The grey dashed line indicates perfect agreement between predicted and true dissociation times.

First we present, for the test set, the comparison of the C–C dissociation times obtained from the *ab initio* molecular dynamics simulations with the dissociation times predicted by the best performing BNN1 and BNN2 models in Fig. 3. Standard deviations of the predicted dissociation time distributions were used to indicate the uncertainty of the BNN model with respect to the predicted dissociation time. We find that BNN can predict dissociation times with high accuracy, despite the medium-sized dataset used for training (10 000 data points from only 250 trajectories). A detailed analysis of the sampling efficiency, *i.e.* the achieved performance with respect to different training set sizes is reported in the ESI.† The prediction accuracy is improved when supplementing the molecular geometry with the velocities of the atoms (Fig. 3B): the mean absolute deviation (MAD) is 2.40 fs for BNN2 while it is 6.55 fs for BNN1. This observation, combined with the observation that prediction accuracies on the validation set are similar for BNN1 or BNN2 (see ESI†) indicates that supplementing geometries with velocities improves the generalisation abilities of the BNN models. In addition, the BNN models generally predict dissociation times with higher uncertainties when the deviation between the predicted and the true dissociation time is larger. We note that the prediction accuracy can likely be improved when supplementing the presented model training procedure with strategies like cross-fold validation for an effective increase of the training set size as proposed in other studies,^42^,^43^ or testing different models such as kernel-based methods.^24^,^44^,^45^ However, in this study we aim to focus on the interpretability of the trained models and *r*^2^ = 0.97 for BNN2 is considered to be sufficient for this purpose.

Once trained, machine learning models can be extensively used to predict the targeted values with high accuracy and low computational cost. As a reference, it takes on one processor less than a minute to generate a set of 250 initial conditions,^35^ a few seconds to predict the corresponding 250 dissociation times with the trained BNN and approximately 31.5 hours to simulate 160 fs of a single *ab initio* molecular dynamics simulation (for the unmethylated dioxetane, at the level of theory used). Here, in addition, we consider the trained BNN as a result by itself and analyse it to see if physical insight can be gained. In particular, we are interested in understanding the following points: How does the BNN reproduce the C–C dissociation timescales with such a good agreement? What correlations has the BNN identified to achieve high accuracies?

### 3.2 Analysis of the trained models to find correlations

**Fig. 4:**
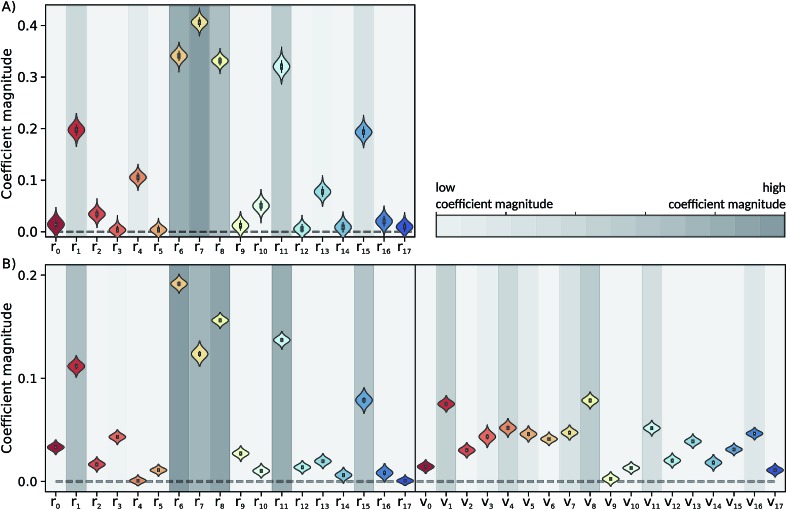
Coefficient magnitude distributions of the input features of the trained (A) BNN1 and (B) BNN2. Input features {*r*_*i*_} and {*v*_*i*_} correspond to geometry coordinates and velocities along normal modes, respectively. The numbering of the normal modes goes from 0 to 17, 0 being the reaction coordinate.

The Laplace distribution (used here as priors for the weights and biases) has been shown to facilitate efficient pruning in BNN parameters,^46^ and is the equivalent to L1-regularization in the Bayesian context.^47^ As such, this choice is well suited to enable the BNN to identify the input features which are most relevant for accurate predictions of the target properties. Generally, the larger the magnitude of the coefficient, the more influential the feature. This relation can be used to determine input features which are relevant for accurate predictions of the dissociation time and therefore relevant to the physical process. Fig. 4 shows the coefficient magnitude distributions of the input features for the Bayesian neural networks BNN1 and BNN2. The numbering of the normal modes goes from 0 to 17, 0 being the reaction coordinate; {*r*_*i*_} correspond to geometry coordinates along these normal modes while {*v*_*i*_} correspond to velocities. As just explained, an input feature that has a large coefficient magnitude does not imply that a distortion along this nuclear coordinate would lead to a longer dissociation time but rather, it indicates that this nuclear coordinate is relevant to predict accurately the dissociation time. It is noted that the choice of input features affects thus not only the learning ability of the BNN, but also the physical interpretation of the correlations that are identified. Trying other input features than the nuclear geometry and velocities in normal mode coordinates is beyond the scope of the present work, but it would be an interesting task to pursue.

Out of the 18 normal modes, BNN1 identifies four as more important for the decomposition reaction: normal modes 6, 7, 8 and 11. They are represented schematically in Fig. 1B. Normal mode 6 corresponds to the (symmetric) stretching of the two C–O bonds, associated with the stretching of the central C–C bond in an out-of-phase manner. Normal mode 7 corresponds to the HCCH dihedral angles. Normal modes 8 and 11 correspond to the stretching of the central C–C bond in phase with symmetric out-of-plane motions of the two OCH_2_ moieties. In BNN2, the coefficients of the initial nuclear geometry (input features *r*_0_ to *r*_17_) are larger in magnitude, in general, than the ones of the initial velocities (input features *v*_0_ to *v*_17_). As in BNN1, the input features *r*_6_, *r*_7_, *r*_8_ and *r*_11_ are identified to be important. In addition, *r*_1_ is also identified to be important. Normal mode 1 corresponds to the OCCO dihedral angle (Fig. 1B). Among the remaining 18 input features corresponding to the initial velocities, the features *v*_1_ and *v*_8_ have the largest coefficient magnitudes. Normal modes 1 and 8 are already identified as important according to the coefficients of the initial position features.

### 3.3 Use of the trained models to test hypothesis

**Fig. 5:**
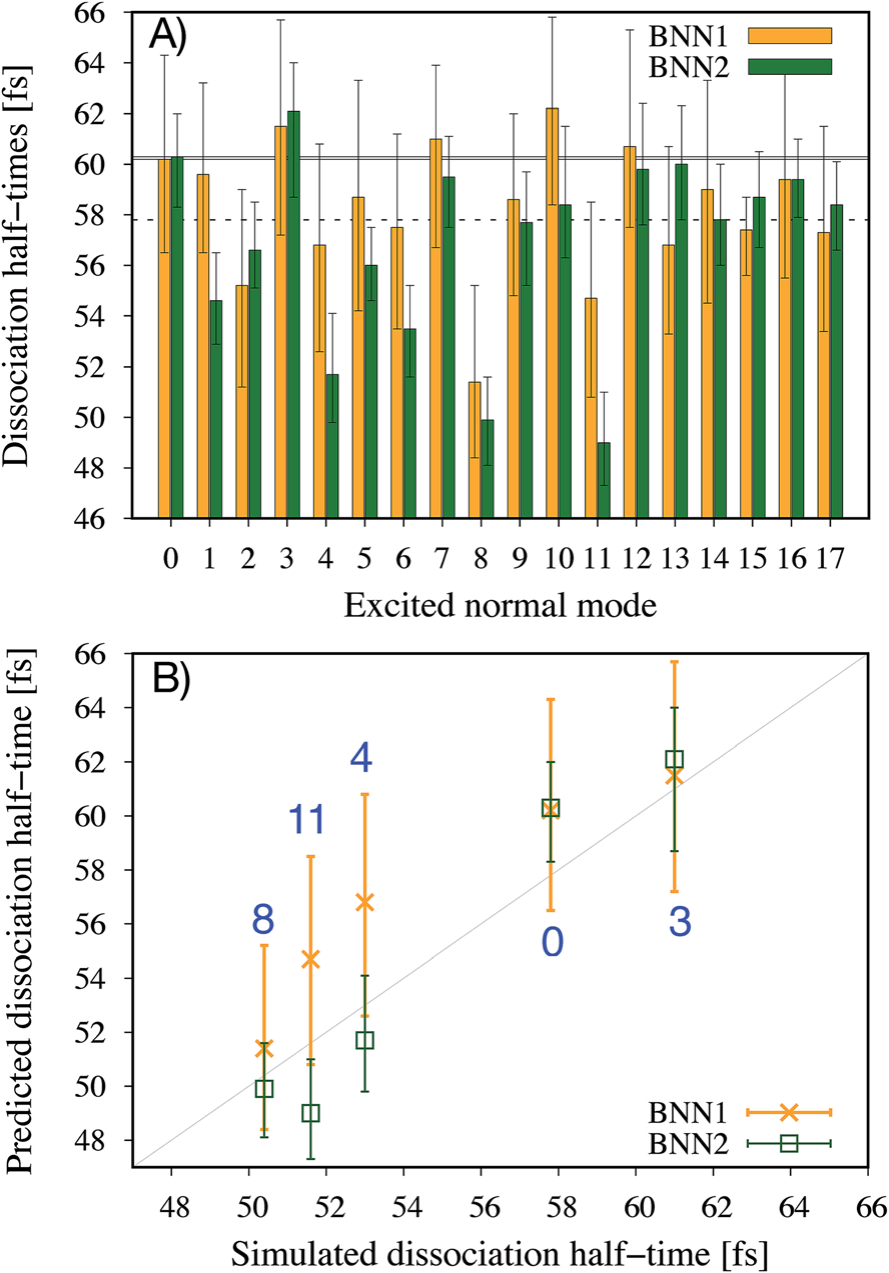
Dissociation half-times for 17 ensembles of 250 initial conditions representing different vibrationally excited states. Ensemble “0” is the reference vibrational ground state. Otherwise, ensemble “*n*” corresponds to a vibrational excitation along normal mode *n*, and ground state along other normal modes. (A) Predicted dissociation half-times by the two trained BNN. The solid and dashed horizontal lines indicate the dissociation time for the ensemble “0”, predicted and extracted from the simulations, respectively. (B) Comparison of the dissociation half-times predicted by the BNN and obtained from *ab initio* molecular dynamics simulations, for five ensembles of 250 trajectories. The error bars represent the 95% confidence intervals of the predictions.

It is noted that the previous analysis, applied on the trained BNN able to make accurate predictions, allows the identification of the most relevant nuclear coordinates for the dissociation dynamics. However, it is not known how these affect the dynamics, *i.e.* whether they make the dissociation occur earlier or later. As an alternative way to gain physical insight, the two trained BNN have been used to predict the dissociation timescales for 17 ensembles of 250 initial conditions, each ensemble representing a vibrational state that is excited to the first level along one particular normal mode. For example, the ensemble “3” corresponds to a vibrational state that is excited along normal mode 3, while it remains in the ground state along all other modes. The ensemble “0” is a reference vibrational ground state along all normal modes, different from the 250 ground state trajectories used for training the BNN. For each ensemble of 250 initial conditions, the corresponding 250 predicted dissociation times are sorted to then extract the overall dissociation half-time, time at which half of the trajectories have dissociated. The predicted dissociation half-times are presented in Fig. 5A: they vary from 49 fs to 63 fs, the reference being 60 fs. It is observed that accelerating the dissociation upon vibrational excitation is more probable than slowing it down, which makes sense since some kinetic energy is added. Excitations of the normal modes 4, 6, 8 and 11 lead to an acceleration of the dissociation while excitation along normal mode 3 is predicted to slow down the dissociation. Normal modes 6, 8 and 11 were already identified as important according to their high coefficient magnitude. Normal mode 3 corresponds to the antisymmetric stretching of the two C–O bonds; normal mode 4 corresponds to the central C–C bond stretching (Fig. 1B). So, according to the trained BNN, in order for the C–C bond to break earlier rather than later, the two formaldehyde moieties need to become planar at the same time as both C–O bonds need to shorten (*i.e.* excitation along normal modes 6, 8 and 11), while if the two C–O bonds stretch in an anti-symmetric fashion (*i.e.* excitation along normal mode 3), this delays dissociation. On the other hand, the high frequency modes (12 to 17) involving the hydrogen atoms seem to affect only mildly the dissociation timescale.

It is interesting to note that, although normal mode 7 is identified as important for predicting accurately the dissociation time (because of its large coefficient magnitude, Fig. 4), vibrational excitation along only this mode seems to barely affect the overall dissociation half-time compared to the reference one (Fig. 5A). To understand this fact, we have used the trained BNN2 to predict the dissociation times for ensembles of initial conditions, where each ensemble now represents a vibrational state that is excited to the first level along two particular normal modes in order to detect second-order effects. The predicted dissociation half-times obtained for each pair of normal modes are given in ESI,† as well as the normal mode z-scores providing information about how excitation along a nuclear coordinate influences the dissociation half-time obtained with an excitation along another coordinate. We find that combining any vibrationally excited normal mode with an excitation along normal mode 7 slows down dissociation. We thus conclude that a sole excitation along normal mode 7 is not sufficient to influence the dissociation timescale (maybe because this mode mainly involves the motion of hydrogen atoms). However, excitation of normal mode 7 in combination with the excitation of another nuclear coordinate could impact the dissociation time significantly. This result would explain the large coefficient magnitude of normal mode 7 in Fig. 4 and also shows how the two proposed methods to gain physical insights from the trained BNN are complementary.

### 3.4 *Ab initio* molecular dynamics simulations of vibrational excited states as “numerical experiments”

To check the accuracy of the dissociation times predicted by the BNN and presented in Fig. 5A, we have simulated the *ab initio* molecular dynamics of four ensembles of 250 trajectories (in addition to the reference ensemble “0”) with excitations along normal modes 3, 4, 8 and 11. It is noted that the *ab initio* molecular dynamics simulations are computationally expensive; they are run here for test purposes, in particular for checking the accuracy of the BNN predictions for initial vibrational excited states not present in the training set.

The four normal modes 3, 4, 8 and 11 were chosen because excitations along modes 8 and 11 are predicted to decrease the most the dissociation half-time, while excitation along mode 3 is predicted to increase it. Normal mode 4 is also predicted to decrease the dissociation half-time and can be seen as the “naive” choice for enhancing C–C bond dissociation without any chemical knowledge, since it corresponds to additional energy along the C–C bond stretching solely. The comparison between the dissociation half-times predicted by the BNN and obtained from the *ab initio* molecular dynamics simulations is presented in Fig. 5B. The qualitative trend is correctly predicted by the BNN. Overall, the errors are on the same order of magnitude as for the reference ensemble “0” although the excited normal modes exhibit larger initial nuclear distortions that may not be present in the training set. In general, BNN2 performs better than BNN1: the root-mean-square deviation is about 1.6 times smaller for the former than for the latter. BNN1 seems to overestimate the dissociation time. An interesting point is that the naive thought that exciting the C–C bond stretching would enhance dissociation is partially validated. It does indeed decrease the dissociation half-time compared to the reference by almost 5 fs, *i.e.* about 8%. However, the BNN successfully find other nuclear modes which decrease the dissociation time further (about 13%) thanks to its finding of physical correlations.

In a previous theoretical study,^11^ the longer dissociation times among the 1,2-dioxetane trajectories were explained by the presence of so-called “frustrated” dissociations – significant stretching of the central C–C bond followed by a shortening rather than a “successful” breaking of the bond. Frustrated dissociations would postpone in time the final successful dissociation. To understand further the link between the predicted/simulated dissociation times and the number of frustrated dissociations, the average number of frustrated dissociations per trajectory are calculated for each ensemble of 250 simulated trajectories (Table 1). As expected, the vibrationally excited ensembles present fewer frustrated dissociations than the reference ground state since more kinetic energy is available to overcome energy barriers. Interestingly, for the four vibrational excited ensembles, the fewer frustrated dissociations, the shorter the dissociation time. This is particularly true for ensemble 8 that exhibits the largest decrease in the average number of frustrated dissociations (35%) and the largest decrease in the dissociation time (13%). The BNN have thus identified nuclear coordinates that would affect the overall dissociation time through the number of these frustrated dissociations. It is noted, however, that ensemble “3” presents a lower average number of frustrated dissociations than the reference ensemble “0” despite its longer dissociation half-time. This indicates that the number of frustrated dissociations is not the only parameter determining the dissociation timescale, dissociations needing to be attempted in order to be frustrated or successful.

**Table 1:** Average number of frustrated dissociations per trajectory for each ensemble of 250 trajectories

Ensemble	0	3	4	8	11
*n*_frus_	1.68	1.52	1.18	1.08	1.17

## 4 Discussion

In this section, the trained models are discussed with first interpreting the identified correlations in the context of chemistry. Finally, implications for chemiexcitation and chemical design are discussed, using the case of the tetramethylated dioxetane molecule.

### 4.1 Interpretation of the findings of the trained models

Interpreting the correlations identified by the BNN leads to the following fundamental concepts known in chemistry: the octet rule,^48^–^50^ the relation between bond order and bond length^51^,^52^ and orbital hybridisation.^53^,^54^ The octet rule states that atoms (with an atomic number *Z* ≥ 5) tend to combine in such a way that each atom has a full valence shell (*i.e.* with eight electrons). This electronic configuration, identical to the one of noble gases, is associated to a maximal stability. As a consequence, the carbon atom that has only four electrons in its valence shell will form four covalent bonds to acquire the four missing electrons. As the C–C bond breaks, each carbon atom has to form a new bond with one of the remaining neighbouring atoms in order to keep eight electrons in the valence shell: it achieves this by forming a double bond with the oxygen atom. The number of bonding electrons between a pair of atoms and thus the bond order determines then the length of a bond. When the single bond between the C and O atoms becomes a double bond, more electrons participate in forming the bond and the bond becomes shorter (*i.e.* excitation along normal mode 6). The molecular shape is also explained with orbital hybridisation, *i.e.* the fact that 2s and 2p atomic orbitals mix, by minimising the repulsion between the pairs of electrons. The theory used to rationalise the molecular shape is known as valence shell electron pair repulsion (VSEPR) model.^55^,^56^ When each carbon atom is surrounded by four atoms (here before dissociation, an oxygen atom, the other carbon atom and two hydrogen atoms), the 2s and three 2p orbitals mix to form the four covalent bonds which adopt a tetrahedral arrangement: this is called sp^3^ hybridisation. It is noted that the tetrahedral conformation of the carbon atoms is constrained in 1,2-dioxetane by the four-membered ring structure. When the C–C bond breaks and each carbon atoms becomes surrounded by only three atoms, then the 2s orbital mixes only with two 2p orbitals to form three sp^2^ hybridised orbitals; the remaining 2p orbital stays intact and will form the π bond by parallel overlap. Minimisation of the repulsion energy is obtained by a trigonal planar geometry. Upon C–C bond breaking, the two formaldehyde moieties become thus planar (*i.e.* excitation along normal modes 8 and 11). In summary, the BNN has identified the following correlation between nuclear coordinates and dissociation time, without any knowledge of electronic structure: the C–C bond breaking must be associated with the formation of a shorter (since double) C–O bond and the planarity of the formaldehyde moieties. This is chemistry!

### 4.2 Implications for chemiexcitation and chemical design

Because the dissociation timescale determines the chemiexcitation yield,^12^ a machine learning model able to find nuclear coordinates that affect efficiently the dissociation dynamics could be used in design of chemiluminescent systems. Here, normal modes 3 and 8 are found to be the nuclear coordinates that induce the largest deviations from the reference ensemble 0 (Fig. 5B). The chemiexcitation yield is actually low in 1,2-dioxetane (0.3%).^57^ A chemical substitution that would enhance the simultaneous planarisation of the two formaldehyde moieties together with the stretching of the central C–C bond would make the dissociation occur earlier and therefore decrease further the chemiexcitation yield. More importantly, a chemical substitution that would induce an asymmetric stretching of the two C–O bonds would make the dissociation occur later and therefore increase the chemiexcitation yield. According to the kinetic model developed in a previous work,^12^ a postponing of the dissociation time of (unmethylated) 1,2-dioxetane by 3.2 fs – as predicted by ensemble 3 compared to the reference ensemble 0 – enhances the chemiexcitation yield by more than a factor of six!

It was observed experimentally that the yield increases by two orders of magnitude upon substitution of the four hydrogen atoms by four methyl groups.^57^ Initially, it was suggested that the greater number of nuclear coordinates in the methyl-substituted compounds would increase the depth of the “entropic trap” and thus the chemiexcitation yield.^9^ Recently, the *ab initio* molecular dynamics of the methyl-substituted dioxetane has been simulated^12^ although these simulations are more than 20 times more computationally expensive than the ones for the unmethylated compound. This later work demonstrated instead that the increase in chemiexcitation yield is due to a mass effect. When training a BNN based on an ensemble of trajectories of the tetramethylated dioxetane, we found that the nuclear coordinates presenting large coefficient magnitudes are the stretching of the C–O bonds, the stretching of the central C–C bond, a planarisation of the two ketone moieties and the O–C–C–O dihedral angle. These coordinates are the equivalent of the normal modes 1, 6, 8 and 11 in the unsubstituted 1,2-dioxetane molecule, which were identified as important for predicting the dissociation time of the unsubstituted dioxetane. This result confirms the similarity in the dissociation dynamics of the unsubstituted and methyl-substituted molecules and therefore that the importance of the methyl groups for increasing the chemiexcitation yield is not the additional nuclear coordinates, but the heavier mass. It also shows how machine learning models help interpreting the results of molecular dynamics simulations.

## 5 Conclusion

Bayesian neural networks have been optimised to predict a specific outcome of an *ab initio* molecular dynamics simulation: the dissociation time of the unmethylated and tetramethylated 1,2-dioxetane molecules from just the initial nuclear geometry (and velocities). This means replacing the *ab initio* molecular dynamics simulation in total and not only the calculation of the potential energy at every time step. Despite the medium size of the dataset used for training, a high prediction accuracy is obtained. More important than the ability to predict a number, we have analysed the trained Bayesian neural networks and demonstrated that machine learning models can help extracting conceptual information from the large amount of data produced by the simulations. Indeed, in order to make accurate predictions, the Bayesian neural networks have figured out that an earlier dissociation must be associated with the planarisation of the two formaldehyde moieties and the symmetric shortening of the C–O bonds. This is in connection with the octet rule, the relation between bond order and bond length and orbital hybridisation, rules that are part of today's common knowledge of chemists. The present work is a step towards achieving one of the grand challenges in the 21st century^1^ and opens thus the way for breakthroughs in chemistry where humans, inspired by the findings of machines, would develop new concepts.

## Conflicts of interest

There are no conflicts to declare.

## Supplementary Material

Supplementary information
