# Supplementary material for: How machine learning can assist the interpretation of ab initio molecular dynamics simulations and conceptual understanding of chemistry
Source: Chem Sci. 2018 Dec 21;10(8):2298–307. doi: 10.1039/c8sc04516j (PMC6385677; doi:10.1039/c8sc04516j)
Supplement: Supplementary file 1 [file SC-010-C8SC04516J-s001.pdf]

Electronic Supplementary Material (ESI) for Chemical Science.
This journal is © The Royal Society of Chemistry 2018

Supplementary Information: How machine learning can assist the interpretation of ab initio molecular dynamics simulations and conceptual understanding of chemistry

Florian Häse,[†] Ignacio Fdez. Galván,[‡] Alán Aspuru-Guzik,[¶,§,∥] Roland Lindh,[‡] and Morgane Vacher[*,‡]

†*Department of Chemistry and Chemical Biology, Harvard University, Cambridge, Massachusetts 02138, United States*

‡*Department of Chemistry – Ångström, The Theoretical Chemistry Programme, Uppsala University, Box 538, 751 21 Uppsala, Sweden*

¶*Department of Chemistry and Department of Computer Science, University of Toronto, Toronto, Ontario M5S 3H6, Canada*

§*Vector Institute for Artificial Intelligence, Toronto, Ontario, M5S 1M1, Canada*

∥*Canadian Institute for Advanced Research (CIFAR), Senior Fellow, Toronto, Ontario, M5S 1M1, Canada*

E-mail: morgane.vacher@kemi.uu.se

Introduction to Principal Component Analysis (PCA)

Principal component analysis (PCA) describes an unsupervised learning strategy in machine learning which operates on an unlabelled dataset. Such datasets only contain parameter vectors, commonly referred to as features, and do not include any associated target values as it is the case in supervised learning problems. PCA analyzes these features with the goal of identifying an orthogonal transformation which projects features onto a set of mutually uncorrelated variables, referred to as the principal components. The principal components are constructed such that the first principal component accounts for as much variability of the dataset as possible. Each next principal component is then constructed to explain the largest possible portion of the remaining variability while satisfying the constraint of being orthogonal to all already identified principal components. Consequently, principal components fully describe the variability in the dataset.

Principal components can be constructed by computing the covariance matrix of all features across the dataset. The covariance matrix can be diagonalized, and the eigenvectors of the covariance matrix present the principal components. The principal component associated with the largest eigenvalue of the covariance matrix accounts for the most variability in the dataset.

PCA can be used as a tool for dimensionality reduction. By construction, the first principal components account for the most variability in the dataset. In practice, it is often found that the contribution of some of the later constructed principal components to the overall variability in the dataset is negligible. Most of the variability, and thus the information content in the dataset, can be explained by the first few principal components. The dimensionality of the dataset can then be reduced by discarding the uninformative principal components.

In this study, we use PCA to determine a statistically diverse set of molecular frames to construct a training set for the Bayesian neural network models. Molecular frames were projected onto the principal components obtained from the covariance matrix constructed

for the combined training and validation set. Within the reduced PCA space representation, we determine the most diverse set of projected features while neglecting the principal components which contribute minimally to the overall variability of the dataset, following the procedure outlined in a previously published work.[1]

Further details on the Bayesian Neural Networks (BNN)

Bayesian neural networks (BNN) represent probabilistic models containing parameters which are modelled as random variables. The output of the BNN is therefore a probability distribution, which can be optimised to resemble a target probability distribution. For the prediction of 1,2-dioxetane dissociation times we chose to model the probability distributions of weights and biases as Laplace distributions $\mathcal{L}$ parametrised with a location $\boldsymbol{\mu}$ and a scale $\boldsymbol{\sigma}$, i.e.

$$\boldsymbol{w}_i \sim \mathcal{L}(\boldsymbol{\mu}_i, \boldsymbol{\sigma}_i), \qquad \boldsymbol{b}_j \sim \mathcal{L}(\boldsymbol{\mu}_j, \boldsymbol{\sigma}_j), \tag{1}$$

where $\boldsymbol{w}_i$ and $\boldsymbol{b}_j$ represent the collection of all weights and biases of all neurons in the BNN.

In addition to the model parameters $\boldsymbol{w}_i$ and $\boldsymbol{b}_j$ the BNN models can differ in their architecture, i.e. the number of neuron layers, the number of neurons per layer and the activation function of the neurons. The optimal choice for these parameters yielding the models with the most accurate predictions was determined from a random grid search. Lower and upper bounds as well as the steps in each hyperparameter for the construction of the hyperparameter grid are reported in table S1. In addition to the model hyperparameters we also benchmarked the effect of the initial learning rate η of the Adam optimisation algorithm.

We evaluated a total of 256 BNN models with hyperparameter sets randomly sampled from the grid of hyperparameters for each of the two featurisation methods (geometries only,

Table S1: BNN hyperparameters considered for optimization in a random grid search. The learning rate set for the Adam optimiser is denoted with η.

Model parameter	Lower bound	Upper bound	Step
lg(η)	-4	-1.5	0.125
lg(regularization)	-4	-2	0.125
# layers	3	5	1
# neurons per layer	50	150	4
activation function	Softsign, LeakyReLU, Tanh		

geometries and velocities). The hyperparameter sets yielding the BNN models with the highest prediction accuracies on the validation set are reported in table S2.

Table S2: Hyperparameters of BNN models with the highest prediction accuracies on the validation set out of 256 generated BNN architectures.

Hyperparameter	BNN1	BNN2
lg(η)	-2.25	-2.25
lg(regularization)	-3	-2.5
# layers	4	4
# neurons per layer	130	126
activation function	Leaky ReLU	Leaky ReLU

Figure S1 shows the lowest mean absolute deviations (MAD) between predicted and true dissociation times achieved by BNN models with different architectures on the validation set. We observe preferences for particular hyperparameter values for all varied hyperparameters. In addition, we do not observe a large difference in the lowest prediction errors achieved by BNN models trained on geometries only, or trained on geometries and velocities.

BNN performance and sampling efficiency

In this section we discuss the sampling efficiency in more detail and determine the performance of the BNN models when they are trained on different fractions of the entire training set. Figure S2 illustrates the achieved sampling efficiencies of both models achieved on the test set. We observe that the performance of the models degrades substantially when trained on less than 3000 frames. However, we note that positive R2 values are achieved even when

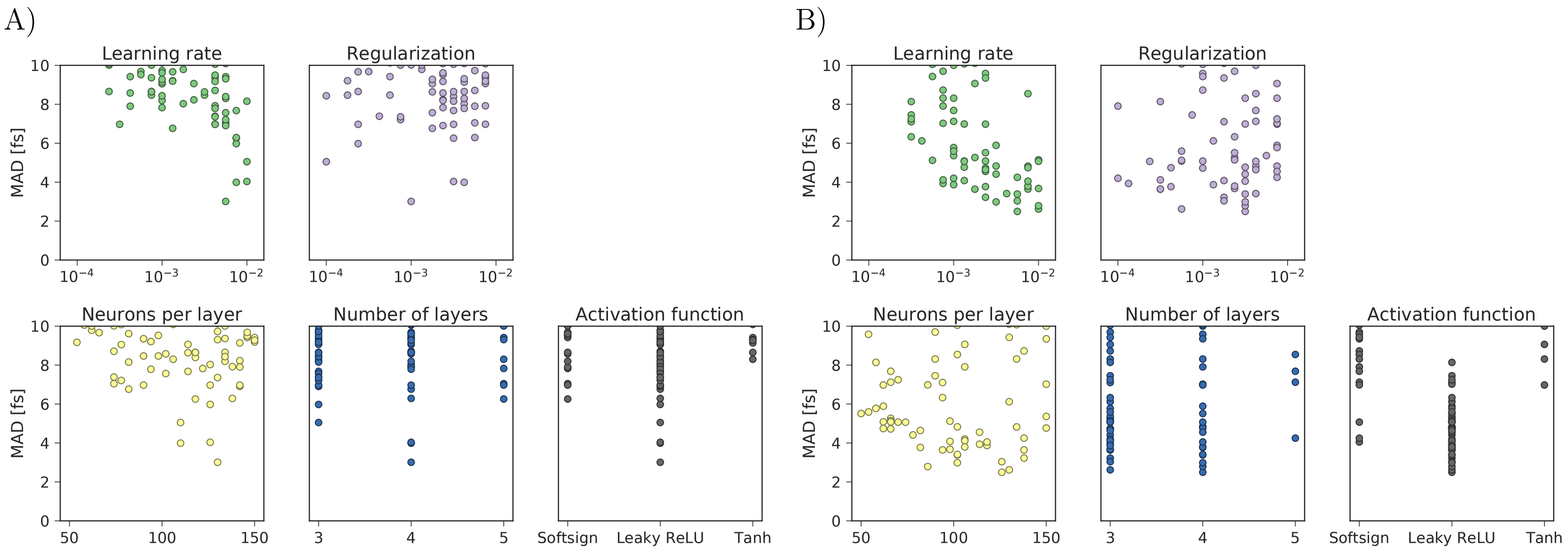

Figure S1: Mean absolute deviations (MAD) achieved by BNN models constructed from different sets of hyperparameters (A) for BNN1 and (B) BNN2.

the size of the training set is reduced by two orders of magnitude.

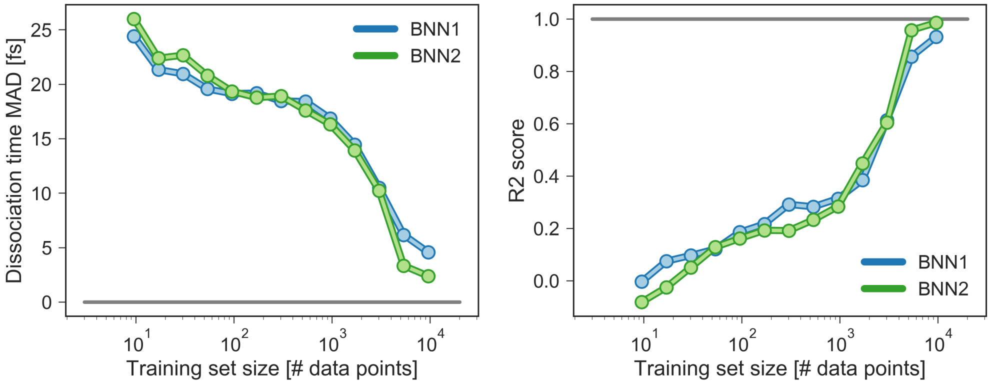

Figure S2: Sampling efficiency of the two trained BNN for different training set sizes. (A): Mean absolute deviation (MAD) achieved on the test set. (B): R2 score achieved on the test set. Dark lines indicate the minimal achievable MAD and maximal achievable R2 score.

Predictions of dissociation half-times for vibrational states excited along two normal modes

We have used the trained BNN2 to predict the dissociation times for 153 ensembles of 250 initial conditions, each ensemble representing a vibrational state that is excited to the first level along two particular normal modes. For example, the ensemble "3,7" corresponds to a vibrational state that is excited along normal modes 3 and 7, while it remains in the ground state along all other modes. The predicted dissociation half-times are given in figure S3, as well as the normal mode z-scores providing information about how a nuclear coordinate influences another one in figure S4. It is noted that positive z-scores indicate that the dissociation is slower. We find that combining any vibrationally excited normal mode with an excitation along normal mode 7 slows down dissociation.

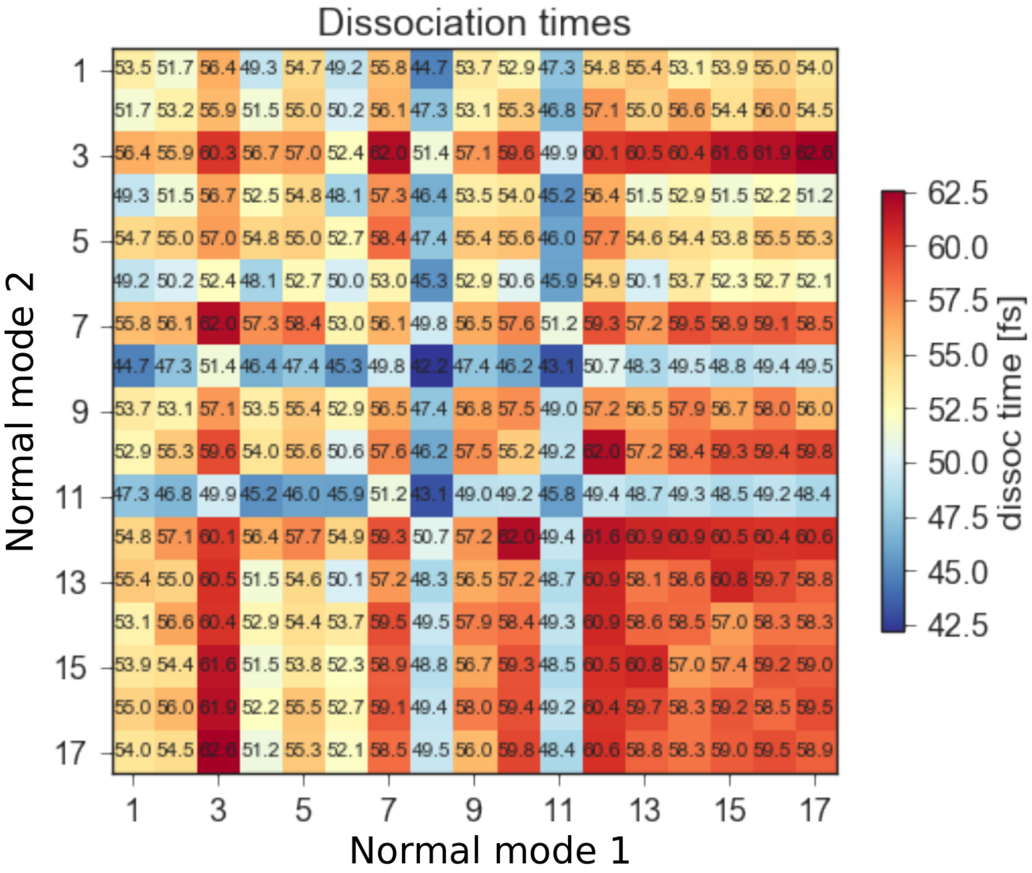

Figure S3: Predicted dissociation half-times for 153 ensembles of 250 initial conditions representing different vibrationally doubly excited states. Ensemble "n, m" corresponds to a vibrational excitation along normal modes n and m, and ground state along other normal modes. By construction the image is symmetric with respect to the (1,1)-(17,17) diagonal.

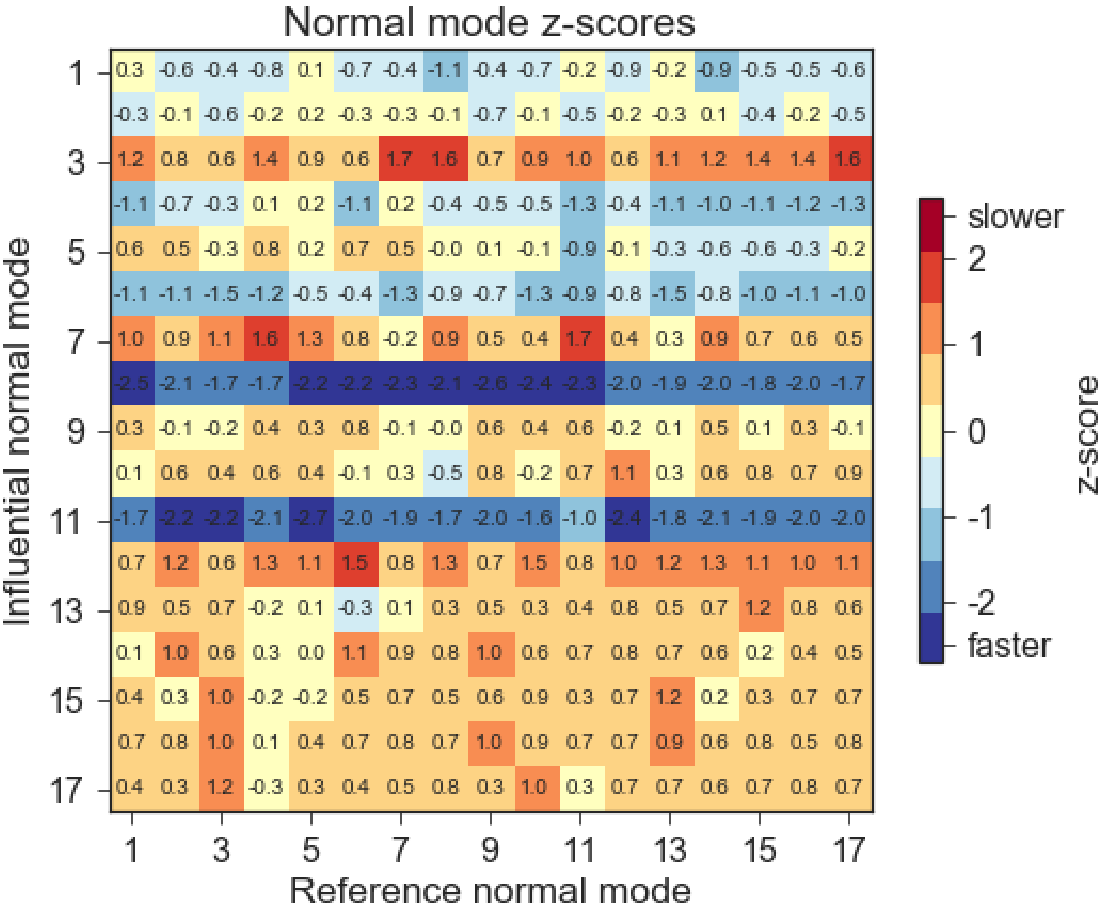

Figure S4: Normal mode z-scores providing information about how excitation along a nuclear coordinate on the y-axis influences the dissociation half-time obtained with an excitation along a reference coordinate on the x-axis.

Dissociation of 1,2-dioxetane from vibrationally excited states

Figure S5 shows the time evolution of the fraction of trajectories that have dissociated for the vibrational ground state (blue), vibrational first excited state along normal mode 3 (red) and along normal mode 8 (purple). The latter two are the nuclear coordinates that induce the largest deviations from the reference ensemble 0.

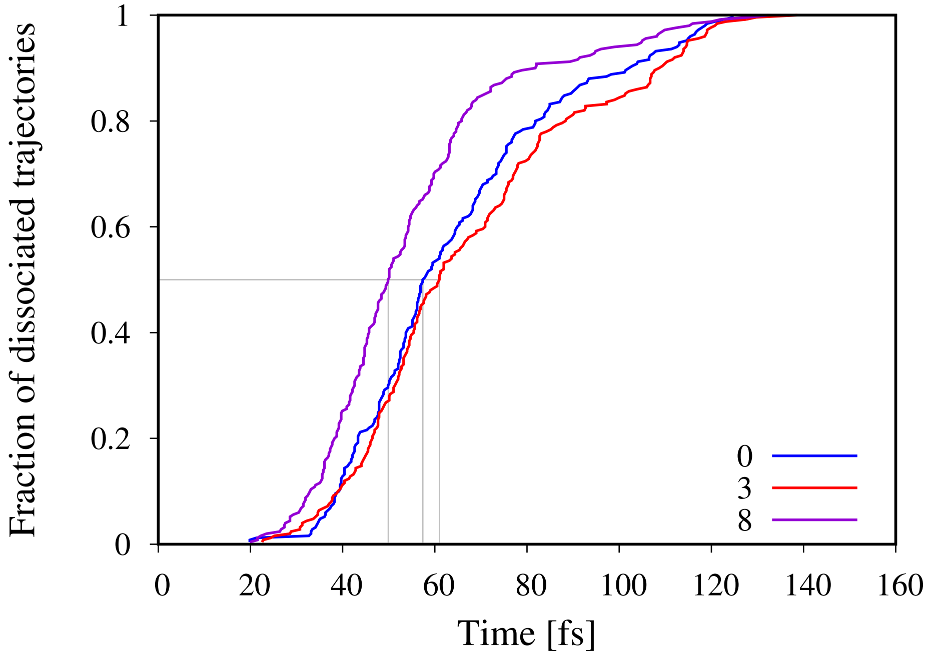

Figure S5: Fraction of trajectories that have dissociated as a function of time for the molecular dynamics simulations for the vibrational ground state (blue), vibrational first excited state along normal mode 3 (red) and vibrational first excited state along normal mode 8 (purple). The thin grey lines indicate the dissociation half-times.

Transition state structure and normal modes of the unmethylated 1,2-dioxetane

The nuclear geometry of the transition state structure for the O–O bond breaking of the unmethylated 1,2-dioxetane is given in Table S3. It was optimised at CASSCF(12,10)/ANO-

RCC-VTZP level of theory using the OpenMolcas package. Tables S4-S8 give the normal modes at the transition state structure, at the same level of theory as for the geometry optimisation.

Table S3: Transition state structure for the O–O bond breaking optimised at CASSCF(12,10)/ANO-RCC-VTZP level of theory [Å].

Atom	x	y	z
C	-0.75188423	0.15702743	-0.62100275
H	-1.33038300	-0.44090287	-1.31225901
H	-0.93985370	1.21031672	-0.77447566
C	0.75188426	-0.15702747	-0.62100257
H	0.93985382	-1.21031676	-0.77447539
H	1.33038305	0.44090285	-1.31225878
O	-1.10698084	-0.22955661	0.71807154
O	1.10698064	0.22955673	0.71807174

References

(1) Häse, F.; Kreisbeck, C.; Aspuru-Guzik, A. Machine learning for quantum dynamics: deep learning of excitation energy transfer properties. *Chem. Sci.* **2017**, *8*, 8419–8426, DOI: 10.1039/C7SC03542J.

Table S4: Normal modes calculated at the transition state structure for the O–O bond breaking, at CASSCF(12,10)/ANO-RCC-VTZP level of theory. The numbering goes from 0 to 17, 0 being the reaction coordinate.

Normal mode 0	x	y	z
C	0.00468	-0.00734	-0.04412
H	-0.04500	-0.03812	0.02697
H	-0.01614	-0.01384	-0.05648
C	-0.00464	0.00730	-0.04422
H	0.01639	0.01386	-0.05651
H	0.04481	0.03828	0.02683
O	0.16239	0.04055	0.03499
O	-0.16242	-0.04053	0.03502
Normal mode 1	x	y	z
C	-0.03040	-0.09645	-0.01998
H	0.08474	-0.29834	0.05787
H	-0.18529	-0.15270	-0.21503
C	0.03015	0.09647	-0.01977
H	0.18549	0.15277	-0.21454
H	-0.08473	0.29844	0.05832
O	-0.04434	0.08881	0.02476
O	0.04452	-0.08884	0.02481
Normal mode 2	x	y	z
C	0.10140	-0.02215	0.08021
H	-0.03383	0.12363	0.06931
H	0.26107	0.01338	0.13858
C	0.10152	-0.02202	-0.08043
H	0.26136	0.01348	-0.13842
H	-0.03356	0.12429	-0.06883
O	-0.09056	0.00796	0.05687
O	-0.09034	0.00786	-0.05675
Normal mode 3	x	y	z
C	0.04902	0.06188	-0.03775
H	0.32237	-0.21349	-0.02418
H	-0.29156	-0.01908	-0.13061
C	0.04822	0.06193	0.03866
H	-0.29189	-0.01868	0.12989
H	0.32018	-0.21183	0.02521
O	-0.03816	-0.03176	0.08442
O	-0.03852	-0.03195	-0.08513

Table S5: ...following of Table S4.

Normal mode 4	x	y	z
C	0.12827	-0.02857	0.08340
H	0.33154	-0.10398	-0.01899
H	0.10691	-0.04721	-0.02021
C	-0.12862	0.02821	0.08254
H	-0.10367	0.04735	-0.01981
H	-0.33365	0.10498	-0.02008
O	0.02617	0.01784	-0.06015
O	-0.02598	-0.01764	-0.05936
Normal mode 5	x	y	z
C	0.01868	0.01357	0.12968
H	0.08141	-0.22385	0.27274
H	-0.22612	-0.05688	-0.06852
C	0.01790	0.01403	-0.13140
H	-0.22849	-0.05685	0.06918
H	0.07997	-0.22481	-0.27577
O	-0.00478	0.00728	-0.07627
O	-0.00418	0.00745	0.07771
Normal mode 6	x	y	z
C	-0.08697	0.01295	0.10473
H	-0.18116	-0.06584	0.24385
H	-0.13634	-0.00222	0.01259
C	0.08720	-0.01295	0.10287
H	0.13374	0.00135	0.01281
H	0.18080	0.06344	0.23970
O	0.03177	0.01911	-0.09456
O	-0.03176	-0.01891	-0.09326
Normal mode 7	x	y	z
C	-0.03992	-0.10831	0.01464
H	-0.01699	0.22377	-0.29166
H	0.07217	-0.03206	0.39945
C	0.03977	0.10858	0.01490
H	-0.07025	0.03246	0.40063
H	0.01772	-0.22464	-0.29185
O	0.00896	0.04069	-0.01776
O	-0.00902	-0.04086	-0.01805

Table S6: ...following of Table S5.

Normal mode 8	x	y	z
C	-0.07908	0.07558	0.01860
H	0.43370	-0.04556	-0.30848
H	-0.24463	0.04637	0.00703
C	0.07895	-0.07593	0.01802
H	0.24363	-0.04663	0.00460
H	-0.43093	0.04579	-0.30616
O	0.01535	0.00176	0.00519
O	-0.01536	-0.00149	0.00533
Normal mode 9	x	y	z
C	-0.01504	-0.05247	0.03967
H	0.31081	0.07358	-0.34386
H	-0.19644	-0.04071	0.39624
C	-0.01576	-0.05161	-0.03962
H	-0.19812	-0.04033	-0.39529
H	0.31357	0.07310	0.34532
O	0.00429	0.03705	-0.01003
O	0.00433	0.03690	0.00984
Normal mode 10	x	y	z
C	-0.06655	0.01314	0.06873ă
H	0.32157	-0.03859	-0.20628
H	0.36868	0.03735	-0.31895
C	-0.06585	0.01327	-0.06805
H	0.36597	0.03787	0.31071
H	0.32385	-0.04252	0.20467
O	0.00622	-0.00990	0.00603
O	0.00615	-0.00954	-0.00592
Normal mode 11	x	y	z
C	-0.07722	-0.02630	0.03484
H	0.05954	0.02296	-0.12280
H	0.49646	0.02460	-0.35715
C	0.07792	0.02615	0.03568
H	-0.49982	-0.02475	-0.36178
H	-0.06271	-0.02315	-0.12531
O	0.00827	-0.01243	0.00400
O	-0.00839	0.01257	0.00402

Table S7: ...following of Table S6.

Normal mode 12	x	y	z
C	0.03191	-0.02120	0.04162
H	-0.12986	0.40093	-0.20942
H	-0.27756	-0.12343	-0.36856
C	0.03272	-0.02128	-0.04222
H	-0.28340	-0.12560	0.37439
H	-0.13219	0.40723	0.21146
O	0.00167	-0.00165	0.00478
O	0.00169	-0.00171	-0.00483
Normal mode 13	x	y	z
C	0.03678	-0.01879	0.04399
H	-0.14520	0.41095	-0.18981
H	-0.29248	-0.12429	-0.36571
C	-0.03552	0.01833	0.04374
H	0.28440	0.12185	-0.36258
H	0.14027	-0.40419	-0.18880
O	0.00258	-0.00180	0.00196
O	-0.00271	0.00186	0.00196
Normal mode 14	x	y	z
C	-0.02860	0.01316	-0.03026
H	0.25995	0.28564	0.31889
H	0.07235	-0.44321	0.05285
C	-0.02959	0.01426	0.03116
H	0.07594	-0.46456	-0.05556
H	0.26728	0.29376	-0.32763
O	0.00054	0.00006	-0.00135
O	0.00055	0.00006	0.00140
Normal mode 15	x	y	z
C	0.02886	-0.02386	0.02756
H	-0.23157	-0.25157	-0.27757
H	-0.08861	0.53118	-0.06719
C	-0.02772	0.02360	0.02634
H	0.08626	-0.51578	-0.06541
H	0.22074	0.23954	-0.26475
O	-0.00009	-0.00000	0.00106
O	0.00007	-0.00001	0.00103

Table S8: ...following of Table S7.

Normal mode 16	x	y	z
C	-0.01732	-0.05817	-0.02347
H	0.27909	0.28862	0.33458
H	-0.07457	0.40577	-0.05736
C	0.01741	0.05795	-0.02352
H	0.07389	-0.40269	-0.05696
H	-0.27957	-0.28907	0.33527
O	-0.00031	-0.00000	0.00014
O	0.00031	0.00000	0.00012
Normal mode 17	x	y	z
C	0.01232	0.06148	0.01780
H	-0.24153	-0.24624	-0.28491
H	0.09171	-0.48134	0.06565
C	0.01246	0.06154	-0.01797
H	0.09160	-0.48052	-0.06563
H	-0.24308	-0.24790	0.28665
O	0.00019	-0.00028	0.00005
O	0.00020	-0.00028	-0.00004
